# Ứng Dụng Kỹ Thuật Giải Trình Tự Gene Thế Hệ Mới Trong Chẩn đoán Lao Kháng Thuốc Trực Tiếp Từ Mẫu đờm Tại Bệnh Viện Phổi Trung ương Năm 2020 – 2022

**DOI:** 10.51403/0868-2836/2023/1466

**Published:** 2024-03-20

**Authors:** Khiếu Thị Thúy Ngọc, Nguyễn Văn Hưng, Trần Huyền Trang, Đinh Thị Hương, Lê Thị Nam, Vũ Ngọc Trung, Đoàn Thu Hà, Nguyễn Văn Khiêm, Nguyễn Kim Cương, Nguyễn Bình Hòa, Đinh Văn Lượng, Nguyễn Thụy Thương Thương, Timothy M Walker, Guy Thwaites

**Affiliations:** 1 Bệnh viện Phổi Trung ương (NLH), Hà Nội; 2 Trường Đại học Y Dược, Đại học Quốc gia Hà Nội (VNU-UMP); 3 Bệnh viện Phổi Hà Nội (HLH); 4 Đơn vị Nghiên cứu Lâm sàng Đại học Oxford (OUCRU), Thành phố Hồ Chí Minh

**Keywords:** *Mycobacterium tuberculosis*, giải trình tự toàn bộ hệ gen, lao đa kháng thuốc (MDR-TB), *Mycobacterium tuberculosis*, mutations, sequencing, multi-drug resistant tuberculosis (MDR-TB)

## Abstract

Việc xác định nhanh chóng tình trạng kháng thuốc kháng sinh là điều cần thiết để điều trị hiệu quả bệnh lao. Nghiên cứu này nhằm mục tiêu dự báo tính kháng thuốc thông qua giải trình tự hệ gene (GTT gene) xác định tính kháng thuốc vi khuẩn lao từ 100 mẫu đờm của bệnh nhân lao có kết quả xét nghiệm Xpert MTB dương tính kháng RIF đến khám tại Bệnh viện (BV) Phổi Trung ương và BV Phổi Hà Nội từ 2020 – 2022. Sau khi loại các mẫu không đạt chất lượng, còn lại 38 mẫu GTT gene có kết quả kiểm soát chất lượng (QC) lớn hơn hoặc bằng 80% với đầy đủ kết quả kháng thuốc kiểu hình và kiểu gen để phân tích đánh giá hiệu quả chẩn đoán. Xét nghiệm GTT gene trực tiếp từ mẫu đờm phát hiện kháng thuốc có độ nhạy và độ đặc hiệu với từng thuốc lần lượt là Isoniazid 91,7% - 100%; Rifampicin 86,79% - 25,0%; Ethambutol 89,5% - 68,4%; Pyrazinamide 56,5% - 100%; Streptomycin 67,7% - 85,7%. Moxifloxacin 50,0% - 96,4%. Xét nghiệm giải trình tự toàn bộ hệ gen từ mẫu đờm cho thấy hiệu quả trong việc ứng dụng công nghệ sinh học phân tử hiện đại vào chẩn đoán sớm bệnh lao kháng thuốc, tiến tới cá thể hóa phác đồ điều trị phù hợp với từng bệnh nhân, tăng tỷ lệ điều trị thành công bệnh lao kháng thuốc tại Việt Nam.

## Abstract (en)

Rapid identification of antibiotic resistance is essential for effective treatment of Tuberculosis (TB). This study aims to predict drug resistance through whole genome sequencing (WGS) to determine drug resistance in *Mycobacterium tuberculosis* from 100 sputum samples of TB patients with Xpert MTB positive and Rifampicin resistant to examined at the Central Lung Hospital and Hanoi Lung Hospital from 2020 - 2022. After eliminating samples that did not meet quality assurance, the remaining 38 samples which had quality control (QC) results greater than or equal to 80% and this sample had complete phenotypic and genotypic drug resistance results to analyze and evaluate diagnostic effectiveness. WGS directly from sputum samples to detect drug resistance has sensitivity and specificity for each drug respectively of Isoniazid 91.7% and 100%; Rifampicin 86.79% and 25.0%; Ethambutol 89.5% and 68.4%; Pyrazinamide 56.5% and 100%; Streptomycin 67.7% and 85.7%. Moxifloxacin 50.0% and 96.4%. Whole-genome sequencing testing from sputum samples shows the effectiveness in applying modern molecular biology technology to early diagnosis of drug-resistant tuberculosis, moving towards individualizing treatment regimens suitable for each individual. patients, increasing the rate of successful treatment of drug-resistant tuberculosis in Vietnam.

## I ĐẶT VẤN ĐỀ

Bệnh lao là một bệnh truyền nhiễm do vi khuẩn *Mycobacterium tuberculosis* gây ra. Hiện nay tỷ lệ nhiễm lao được ước tính là 1/3 dân số thế giới, khoảng 10,6 triệu người mắc lao mới. Sáu triệu đàn ông, 3,4 triệu phụ nữ và 1,2 triệu trẻ em, hơn 1,6 triệu người chết vì bệnh lao vào năm 2021. Trên toàn thế giới, bệnh lao là nguyên nhân gây tử vong đứng thứ 13 và là nguyên nhân truyền nhiễm gây tử vong đứng thứ hai sau COVID-19 (trên HIV và AIDS). Bệnh lao hiện diện ở mọi quốc gia và mọi lứa tuổi. Nhưng bệnh lao có thể chữa được và phòng ngừa được [1].

Bệnh lao đang trở nên nghiêm trọng hơn với sự xuất hiện các chủng lao kháng đa thuốc (Multi-Drugs Resistant – MDR TB), tức là thể lao với vi khuẩn kháng ít nhất hai loại thuốc chống lao mạnh nhất là isoniazid (INH) và rifampicin (RMP). Theo báo cáo của Tổ chức Y tế Thế giới năm 2022, gánh nặng bệnh lao kháng thuốc (DR-TB) cũng được ước tính tăng lên từ năm 2020 đến năm 2021, với 450 000 trường hợp bệnh nhân mới. Số người được điều trị bệnh lao RR-TB và bệnh lao đa kháng thuốc (MDR-TB) đã giảm từ năm 2019 đến năm 2020. Số người được báo cáo bắt đầu điều trị bệnh lao RR-TB và MDR-TB vào năm 2021 là 161.746 người, bao gồm chỉ khoảng một phần ba số người có nhu cầu. Tỷ lệ điều trị thành công bệnh lao kháng thuốc là 60% trên toàn cầu vẫn ở mức thấp [2].

Việt Nam thuộc nhóm 30 quốc gia có gánh nặng bệnh lao và lao kháng thuốc cao nhất thế giới. Năm 2022, ước tính có 172 000 người mắc lao với khoảng 13 000 ca tử vong. Số mắc lao kháng rifampicin hoặc đa kháng thuốc khoảng 8.200 trường hợp [3]. Hướng tới mục tiêu thanh toán bệnh lao vào năm 2035, Việt Nam đang tiến đến áp dụng công nghệ giải trình tự toàn bộ hệ gen vi khuẩn *M. tuberculosis* phát hiện gen đột biến để chẩn đoán lao kháng thuốc, từ đó tối ưu hóa phác đồ điều trị cho từng bệnh nhân lao [1]. Vì thế hiểu biết về đặc điểm phân tử quy định tính kháng thuốc ở quần thể vi khuẩn gây bệnh lao là nền tảng quan trọng cho việc áp dụng thành công công nghệ này ở quy mô quốc gia. Hiện nay việc xác định vi khuẩn lao và tính kháng thuốc dựa vào phương pháp nuôi cấy vi khuẩn và làm kháng sinh đồ trên môi trường lỏng MGIT, bên cạnh đó việc ứng dụng sinh học phân tử cũng đang tạo ra những đột phá trong phát hiện vi khuẩn lao kháng thuốc. Tuy nhiên, các phương pháp này vẫn còn những hạn chế về thời gian xét nghiệm kéo dài đối với nuôi cấy hoặc sinh học phân tử test nhanh thì chỉ chẩn đoán tính kháng một số thuốc như Xpert MTB/RIF, LPA hàng 1, 2. Cần thiết phát triển những kỹ thuật mới, công nghệ mới như giải trình tự gen thế hệ mới (NGS) cho phép dự báo tính kháng thuốc nhanh hơn chỉ trong vài ngày với độ nhạy và độ đặc hiệu cao trực tiếp từ mẫu lâm sàng.

Các nghiên cứu về sinh học phân tử trong chẩn đoán lao kháng thuốc đã cho thấy cơ chế kháng thuốc ở vi khuẩn lao là do trong quá trình tiến hóa, có các đột biến xuất hiện trong một số gen chức năng quy định tính kháng thuốc tương ứng. Các chủng vi khuẩn lao kháng đa thuốc (kháng isoniazid và rifampicin) là do có liên quan tới đột biến tại codon 315 trên gen katG và đột biến ở một vùng ngắn gồm 27 codon nằm gần trung tâm của gen rpoB. Trong khi việc phát hiện tính kháng RIF cơ bản chỉ cần khảo sát ở vùng hot spot (81 bp) tại vùng RRDR của gen rpoB, thì việc phát hiện tính kháng đối với các loại thuốc hiện có và các loại thuốc mới sẽ cần được nghiên cứu sâu hơn về sinh học phân tử của hơn 90 gen trong toàn bộ bộ gen của chủng MTBC dài hơn 4 triệu base [4, 5]. Do đó, nghiên cứu nhằm mục tiêu dự báo tính kháng thuốc thông qua giải trình tự hệ gene (GTT gene) xác định tính kháng thuốc vi khuẩn lao từ 100 mẫu đờm của bệnh nhân lao có kết quả xét nghiệm Xpert MTB dương tính kháng RIF đến khám tại BV Phổi Trung ương và BV Phổi Hà Nội từ 2020 – 2022.

## II PHƯƠNG PHÁP NGHIÊN CỨU

### 2.1 Đối tượng nghiên cứu

Mẫu vi khuẩn *M. tuberculosis* được phân lập từ bệnh nhân lao có kết quả xét nghiệm Xpert MTB dương tính kháng RIF đến khám tại Bệnh viện Phổi Trung ương và Bệnh viện Phổi Hà Nội trong giai đoạn từ năm 2020 – 2022.

#### Tiêu chuẩn lựa chọn mẫu

Mẫu đờm được thu thập từ bệnh nhân/người nghi lao phổi mới từ 18 tuổi trở lên chưa điều trị với thuốc lao hoặc điều trị lao dưới hoặc bằng 30 ngày.

Mẫu đờm đạt chất lượng và có thông tin đầy đủ đi kèm bao gồm: Họ và tên bệnh nhân, tuổi, giới, khoa, tình trạng điều trị với thuốc lao tại thời điểm thu mẫu, tiền sử bệnh, loại bệnh phẩm.

#### Tiêu chuẩn loại trừ

Mẫu đờm thu thập từ người nghi lao có tiền sử điều trị lao trên 30 ngày hoặc người nghi lao dưới 18 tuổi.

Mẫu đờm không đạt chất lượng, không có thông tin đầy đủ thông tin bao gồm: Họ và tên bệnh nhân, tuổi, giới, khoa, tình trạng điều trị với thuốc lao tại thời điểm thu mẫu, tiền sử bệnh, loại bệnh phẩm.

Mẫu đờm từ người nghi lao không đồng ý tham gia nghiên cứu.

### 2.2 Địa điểm và thời gian nghiên cứu

Nghiên cứu được thực hiện tại Bệnh viện Phổi Trung ương và Bệnh viện Phổi Hà Nội từ tháng 11/2020 đến hết tháng 12/2022.

Địa điểm thực hiện xét nghiệm: Khoa Vi sinh và Labo lao chuẩn Quốc gia, Bệnh viện Phổi Trung ương.

### 2.3 Thiết kế nghiên cứu

Phương pháp nghiên cứu mô tả cắt ngang.

### 2.4 Cỡ mẫu nghiên cứu

Tổng số 100 mẫu đờm của bệnh nhân lao có kết quả xét nghiệm Xpert MTB dương tính kháng RIF đến khám tại Bệnh viện Phổi Trung ương và Bệnh viện Phổi Hà Nội trong giai đoạn từ năm 2020 – 2022.

### 2.5 Phương pháp chọn mẫu

Thu thập toàn bộ các mẫu đờm của bệnh nhân lao thỏa mãn tiêu chí lựa chọn và đồng ý tham gia nghiên cứu.

### 2.6 Biến số nghiên cứu

Nhóm biến số về đặc điểm nhân khẩu họ tên, tuổi, giới tính, tiền sử của bệnh nhân; kết quả xét nghiệm của các chủng vi khuẩn *M. tuberculosis* phân lập được từ mẫu đờm của bệnh nhân theo phân loại nhạy, kháng thuốc, loại gen và vị trí đột biến kháng thuốc.

### 2.7 Phương pháp thu thập thông tin

Đặc điểm nhân khẩu học được thu thập qua phiếu thu thập thông tin của người bệnh ngay từ khi đến khám và thu mẫu xét nghiệm.

Các thông tin về kết quả xét nghiệm được ghi nhận vào sổ xét nghiệm hàng ngày và cập nhật cho nhóm nghiên cứu.

#### Xét nghiệm thực hiện trong nghiên cứu

Mẫu đờm của các bệnh nhân có kết quả xét nghiệm Xpert MTB dương tính kháng RIF được thu thập với số lượng từ 4 - 6ml đờm đặc, nhày mủ. Các mẫu được xử lí mẫu theo quy trình nuôi cấy vi khuẩn lao trên môi trường lỏng MGIT. Nếu kết quả MGIT dương tính sẽ được tiếp tục làm định danh, kháng sinh đồ để xác định tính kháng thuốc hàng 1, 2 và các thuốc khác của vi khuẩn lao phân lập được. Các kỹ thuật xét nghiệm thực hiện theo tài liệu hướng dẫn quy trình thực hành chuẩn các xét nghiệm Vi sinh lao (2018), Bộ Y tế - CTCLQG [5, 6].

Các mẫu đờm bảo quản ở -80°C và thực hiện tách DNA để giải trình tự hệ gene xác định tính kháng thuốc. *Mycobacterium tuberculosis* (MTB) trong mẫu đờm sẽ bị bất hoạt bởi sự kết hợp giữa nhiệt và lysozyme. Thành tế bào vi khuẩn sau đó sẽ bị phá vỡ bằng phương pháp cơ học. DNA sau tách chiết sẽ được tinh sạch bằng hạt AMPure XP và sẵn sàng cho thực hiện giải trình tự gen vi khuẩn lao bằng hệ thống Illumina MiniSeq theo quy trình số WGS 06 – BVPTW. Quá trình giải trình tự kết thúc sẽ xuất các trình tự (read) trong file với định dạng fastq.gz. Kết quả giải trình tự cho biết định danh tên loài vi khuẩn, dòng vi khuẩn và dự báo tính kháng thuốc bằng cách sử dụng phần mềm sinh tin MyKrobe 10.0.

### 2.8 Xử lý và phân tích số liệu

Nhập số liệu vào file excel, sử dụng phần mềm tin sinh Mykrobe 10.0 và thống kê SPSS để phân tích dữ liệu nghiên cứu.

### 2.9 Đạo đức nghiên cứu

Nghiên cứu đã được Hội đồng đạo đức trong Nghiên cứu Y sinh học của Bệnh viện Phổi Trung ương cho phép thực hiện theo quyết định số 21/CT-HDDD ngày 25/5/2020.

## III KẾT QUẢ

### 3.1 Sơ đồ thu mẫu và thực hiện xét nghiệm

Nghiên cứu thu thập 100 mẫu đờm của bệnh nhân lao đạt tiêu chuẩn chọn mẫu đến khám tại Bệnh viện Phổi Trung ương và Bệnh viện Phổi Hà Nội trong giai đoạn từ năm 2020 – 2022. Các mẫu được xử lý đồng nhất, khử tạp và chia làm 2 phần. Một phần được bảo quản ở -80 ^0^ C để thực hiện giải trình tự toàn bộ hệ gen trên hệ thống Miniseq Illumina. Một phần mẫu đờm được nuôi cấy phân lập và thực hiện kháng sinh đồ thuốc lao hàng 1, 2 từ các mẫu cấy dương tính với MTB.

Kết quả nuôi cấy có 95 mẫu dương tính với MTB và được tiếp tục làm kháng sinh đồ chẩn đoán kháng thuốc lao hàng 1, hàng 2. Các mẫu đờm tương ứng với 95 mẫu cấy dương tính với MTB được tách DNA và chạy giải trình tự gene. Trong quá trình tách DNA, nhóm nghiên cứu đã sử dụng 2 loại sinh phẩm tách DNA: (1) Trang thiết bị, sinh phẩm tách DNA của hãng Truenat Molbio. Đây là sinh phẩm tách chiết DNA tự động, trong quy trình thực hiện không có bước khử DNA người, sản phẩm thu được có chất lượng kém, không tinh sạch và bị lẫn nhiều DNA người. Kết quả 18 mẫu đờm có nồng dộ DNA khi đo bằng Qubit dưới 2.0 (ng/ul), không phù hợp cho thực hiện các bước tiếp theo của quy trình giải trình tự gen; (2) Tách DNA bằng sinh phẩm của hãng Qiagen trong đó 3 mẫu có nồng độ DNA thấp dưới 2,0 (ng/ul); 74 mẫu có nồng độ DNA trên 2,0 (ng/ul), độ tinh sạch trong giới hạn đo bằng Nanodrop bước sóng A260/280 từ 1,8 – 2,0 và A260 / 230 trong khoảng 2,0 – 2,2 được tiếp tục thực hiện giải trình tự gen.

Nghiên cứu đã biên soạn và ban hành các quy trình kỹ thuật cho việc triển khai giải trình tự gen bao gồm:

+ Quy trình tách DNA trực tiếp từ mẫu đờm;

+ Quy trình tách DNA từ mẫu chủng cấy MGIT;

+ Quy trình tinh sạch DNA vi khuẩn lao bằng hạt từ tính;

+ Quy trình chạy giải trình tự gen bằng hệ thống Illumina MiniSeg;

+ Quy trình phân tích tin sinh dữ liệu giải trình tự gen.

Kết quả giải trình tự của 74 mẫu đờm có 6 mẫu nhiễm (contaminate) không có dữ liệu về loài và tính kháng thuốc, 30 mẫu có kết quả kiểm soát chất lượng (QC) dưới 80% và không có dữ liệu về loài và tính kháng thuốc, 38 mẫu có QC đạt trên 80%, các thông tin kháng thuốc kiểu gen đầy đủ để phân tích so sách với kết quả kháng thuốc kiểu hình. Kết quả phân tích giá trị chẩn đoán của xét nghiệm giải trình tự toàn bộ hệ gen vi khuẩn lao trực tiếp từ mẫu đờm được thể hiện ở bảng 1 và 2.

### 3.2 Giá trị chẩn đoán của xét nghiệm giải trình tự gen từ mẫu đờm

Phân loại kháng thuốc kiểu hình và kháng thuốc kiểu gen của 38 mẫu nghiên cứu được thể hiện ở bảng 1. Các kiểu kháng thuốc (R – Resistant) hoặc nhạy cảm thuốc (S – Sensitive) được xác định bằng kháng sinh đồ dựa trên nuôi cấy trên môi trường lỏng (Mycobacteria_ growth_indicator MGIT) tại khoa Vi sinh và Labo Lao chuẩn Quốc gia, Bệnh viện Phổi Trung ương. Kết quả cho thấy tỷ lệ chủng kháng của Isoniazid là 36/38 (94,7%); Rifampicin là 30/38 (78,9%); Ethambutol 19/38 (50,0%); Pyrazinamid 23/38 (60,5%); Streptomucin 31/38 (81,6%); Moxifloxacin 10/38 (26,3%).

Kết quả ở bảng 2 cho thấy độ nhạy (Se), độ đặc hiệu (Sp), giá trị dự báo dương tính (PPV), giá trị dự báo âm tính (NPV) của xét nghiệm giải trình tự gen từ mẫu đờm khi so sánh với kháng sinh đồ kiểu hình của các thuốc lần lượt là: Thuốc Isoniazid độ nhạy 91,7% và độ đặc hiệu 100%. Độ nhạy của xét nghiệm giải trình tự trong chẩn đoán kháng Rifampicin là 86,79% và độ đặc hiệu 25,0%. Ethambutol có độ nhạy 89,5%, độ đặc hiệu 68,4%. Pyrazinamide có độ nhạy 56,5%, độ đặc hiệu 100%. Streptomycin có độ nhạy 67,7%, độ đặc hiệu 85,7%. Moxifloxacin (MFX) có độ nhạy 50,0%, độ đặc hiệu 96,4%.

Khi so sánh giá trị AUC (Area Under The Curve - Khu vực dưới đường cong) cho thấy kỹ thuật giải trình tự gen có ý nghĩa chẩn đoán rất cao với thuốc Isoniazid (0,948); các thuốc có giá trị AUC ở mức trên trung bình là Ethambutol (0,785), Pyrazinamide (0,782), Streptomycin (0,761), Moxifloxacin (0,730). Thuốc có giá trị AUC ở mức trung bình là Rifampicin (0,557).

## IV BÀN LUẬN

Trong nghiên cứu này, chẩn đoán kháng Isoniazid có độ nhạy 91,7% và độ đặc hiệu 100%. Kết quả này rất có ý nghĩa và thống nhất với các công bố của Papaventis và cộng sự [6] khi phân tích về tỷ lệ kháng Isoniazid trong nhóm bệnh nhân có kháng Rifampicin. Kết quả này cũng phù hợp với công bố của WHO khi đưa ra khuyến cáo về giá trị của chẩn đoán bằng giải trình tự gen [7, 8]. Độ nhạy của xét nghiệm giải trình tự toàn bộ hệ gen trong chẩn đoán kháng Rifampicin là 86,7% và độ đặc hiệu là 25%. Như đã đề cập ở trên, toàn bộ 38 mẫu đờm thu nhận có kết quả xét nghiệm Xpert dương tính kháng Rifampicin, so sánh với kết quả kháng thuốc kiểu hình kháng Rifampicin là 30/38 (78,9%). Đối chiếu với tài liệu của WHO 2021 công bố về độ nhạy và độ đặc hiệu của xét nghiệm giải trình tự so với kháng sinh đồ kiểu hình có giá trị tương ứng là 93,8% và 98,2% [7]. Trong nghiên cứu này chúng tôi sử dụng kháng sinh đồ kiểu hình với nồng độ Rifampicin 0,5ug/ml theo hướng dẫn của WHO 2018 [9]. Tuy nhiên nồng độ này thấp hơn nồng độ Rifampicin 1,0ug/ml nêu trong tài liệu cập nhật của WHO 2021 [10]. Điều này cho thấy đối với các mẫu lâm sàng cần xem xét điều chỉnh nồng độ kháng sinh trong việc thực hiện xét nghiệm kháng sinh đồ kiểu hình và cân nhắc trước khi đưa ra phác đồ điều trị phù hợp do kết quả kháng sinh đồ kiểu hình và kiểu gen có sự sai khác đáng kể [11].

Trong chẩn đoán lao kháng Ethambutol, kết quả cho thấy độ nhạy của xét nghiệm giải trình tự gen là 86,7%, trong đó 17/19 mẫu được chẩn đoán kháng Ethambutol bằng xét nghiệm giải trình tự gen so với kháng sinh đồ kiểu hình. Tuy nhiên, độ đặc hiệu của xét nghiệm chỉ đạt 68,4% so với kháng sinh đồ. Đột biến trong các trường hợp này được xác định là embB_M306I, embB_G406A, embB_Q497R, đã được Tổ chức Y tế Thế giới công bố năm 2021 [12].

Độ nhạy và độ đặc hiệu của chẩn đoán kháng Streptomycin là 67,7,3% và 85,7%. Tương tự như với Ethambutol, có 21/31 mẫu được chẩn đoán kháng Streptomycin bằng cả kiểu hình và kiểu gen và giá trị dự báo dương tính của xét nghiệm giải trình tự gen trong chẩn đoán kháng Streptomycin rất cao 95,5% khi so sánh với xét nghiệm kháng sinh đồ kiểu hình.

Chẩn đoán kháng Pyrazinamide và Moxifloxacin có những hạn chế nhất định khi so sánh với kháng sinh đồ kiểu hình. Pyrazinamide có độ nhạy 56,5%, độ đặc hiệu 100%, Moxifloxacin có độ nhạy 50,0% và độ đặc hiệu 96,4%. Công bố của WHO 2021 đối với hai thuốc này thì độ nhạy và độ đặc hiệu cũng thấp hơn so với các thuốc lao hàng 1. Các trình tự và vị trí đột biến gen liên quan đến tính kháng thuốc Fluoroquinolones vẫn đang được WHO xem xét bổ sung và chưa đưa vào danh mục khuyến cáo bổ sung năm 2021 [7].

Trong dữ liệu có sẵn cho phân tích này, không có đột biến nào đáp ứng tiêu chí liên quan đến kiểu hình kháng các thuốc mới như Bedaquiline hoặc Ceftazidime. Điều này không mâu thuẫn với các nghiên cứu trước đây cho thấy atpE và Rv0678 là các gen kháng chính đối với một hoặc cả hai tác nhân. Thay vào đó, kết quả phân tích này có lẽ là do những hạn chế sau. Đầu tiên, hầu hết các đột biến ở Rv0678 và atpE đều hiếm gặp. Bên cạnh đó, một số đột biến Rv0678 dẫn đến MIC gần với nồng độ ngưỡng, do đó kết quả kháng thuốc kiểu hình được phân loại không nhất quán [11].

## V KẾT LUẬN

Trong nghiên cứu này, tỷ lệ giải trình tự hệ gene từ mẫu đờm thành công là 38/95 (40%). Nồng độ DNA cho xét nghiệm giải trình tự hệ gen trên 2,0 (ng/ul), độ tinh sạch trong giới hạn đo bằng Nanodrop bước sóng A260/280 từ 1,8 – 2,0 và A260/230 trong khoảng 2,0 – 2,2.

Kỹ thuật giải trình tự toàn bộ hệ gen *M. tuberculosis* có giá trị cao trong chẩn đoán kháng thuốc lao hàng 1 và hàng 2. Trong đó, việc phát hiện kháng thuốc Isoniazid có độ nhạy 91,7% và độ đặc hiệu 100%. Độ nhạy của xét nghiệm giải trình tự trong chẩn đoán kháng Rifampicin là 86,79% và độ đặc hiệu 25,0%. Ethambutol có độ nhạy 89,5%, độ đặc hiệu 68,4%. Pyrazinamide có độ nhạy 56,5%, độ đặc hiệu 100%. Streptomycin có độ nhạy 67,7%, độ đặc hiệu 85,7%. Moxifloxacin (MFX) có độ nhạy 50,0%, độ đặc hiệu 96,4%.

Nghiên cứu này góp phần cung cấp dữ liệu kháng thuốc của 38 mẫu đờm có kết quả giải trình tự toàn bộ hệ gen, so sánh với kết quả kháng sinh đồ trong nghiên cứu có giá trị chẩn đoán với 6 thuốc lao hàng 1 và hàng 2. Kết quả chưa nhiều nhưng rất có giá trị trong việc ứng dụng thành công xét nghiệm giải trình tự gen trực tiếp từ mẫu đờm cho kết quả kháng thuốc lao hàng 1, 2 cùng lúc trong khoảng từ 3 - 5 ngày làm việc. Trên cơ sở tối ưu hóa quy trình kỹ thuật thực hiện, xét nghiệm giải trình tự toàn bộ hệ gen từ mẫu đờm cho thấy hiệu quả trong việc ứng dụng công nghệ sinh học phân tử hiện đại vào chẩn đoán sớm bệnh lao kháng thuốc, tiến tới cá thể hóa phác đồ điều trị phù hợp với từng bệnh nhân, tăng tỷ lệ điều trị thành công bệnh lao kháng thuốc tại Việt Nam.

### Lời cảm ơn

Nghiên cứu này được thực hiện với sự hỗ trợ kinh phí của nhiệm vụ hợp tác Quốc tế về Khoa học và Công nghệ theo nghị định thư giữa Bệnh viện Phổi Trung ương, Đơn vị Nghiên cứu Lâm sàng Đại học Oxford (OUCRU) Tp Hồ Chí Minh, quỹ MRC Newton của Vương quốc Anh mã số MR/R006319/1 và Bộ Khoa học và Công nghệ, đề tài “Giải trình tự gen vi khuẩn lao nhằm dự báo tính nhạy cảm với thuốc kháng lao ở bệnh nhân lao kháng đa thuốc tại Việt Nam”, mã số đề tài NĐT.81.GB/20. Chủ nhiệm đề tài là PGS. TS. BS. Nguyễn Văn Hưng, Bệnh viện Phổi Trung ương và đồng chủ nhiệm là TS. BS. Guy Thwaites, Đơn vị Nghiên cứu Lâm sàng Đại học Oxford (OUCRU) Tp Hồ Chí Minh.

## Figures and Tables

**Hình 1:**
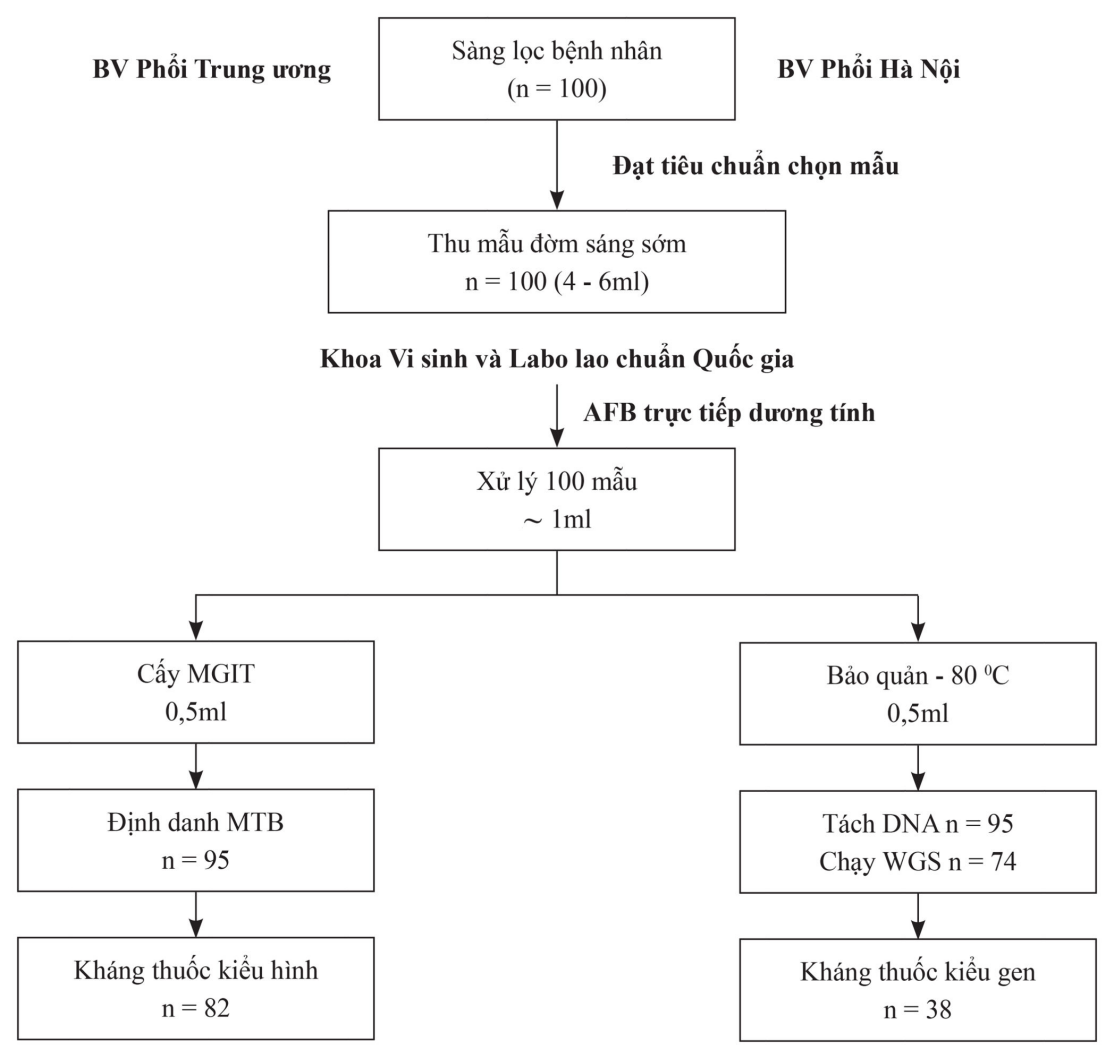
Sơ đồ thu mẫu và thực hiện xét nghiệm

**Hình 2:**
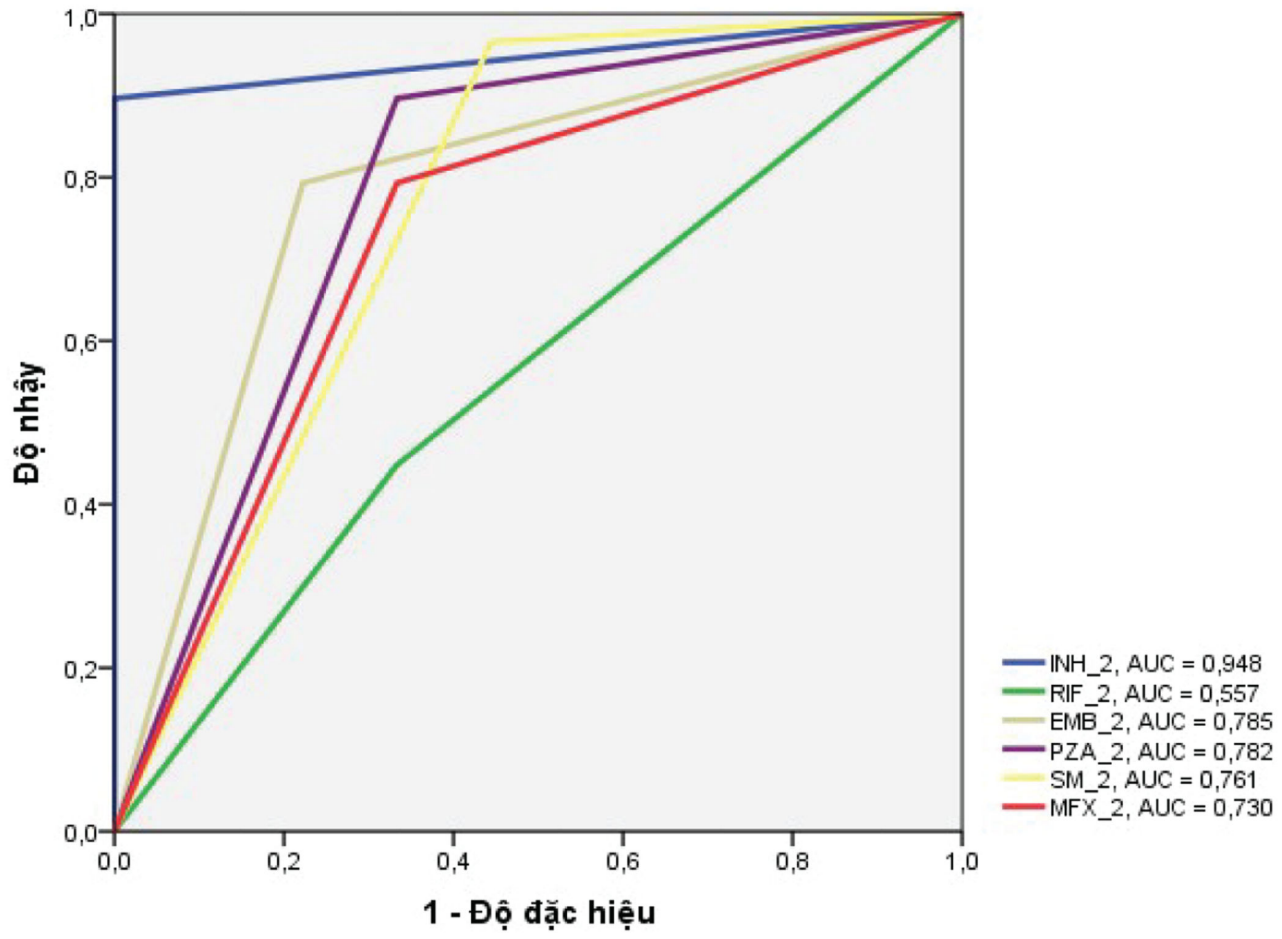
Biểu đồ tương quan ROC của các thuốc lao khi so sánh giữa kết quả kháng thuốc kiểu hình và kiểu gen

**Bảng 1:** Số lượng và tỷ lệ kháng thuốc kiểu hình và kiểu gen

		Kháng thuốc kiểu hinh				Tống số
		R		S		
		n	%	n	%	
Kháng thuốc kiểu gene	Isoniazid					
	R	33	91,7	0	0	33
	S	3	8,3	2	100	5
	Tổng số	36		2		38
	Rifampicin					
	R	26	86,7	6	75	32
	S	4	13,3	2	25	6
	Tổng số	30		8		38
					
	Ethambutol					
	R	17	89,5	6	32	23
	S	2	10,5	13	68	15
	Tổng số	19		19		38
	Pyrazinamide					
	R	13	56,5	0	0	13
	S	10	43,5	15	100	25
	Tổng số	23		15		38
	Streptomycin					
	R	21	67,7	1	14	22
	S	10	32,3	6	86	16
	Tổng số	31		7		38
	Moxifloxacin					
	R	5	50,0	1	4	6
	S	5	50,0	27	96	32
	Tổng số	10		28		38

*R: Resistant – Kháng thuốc; S: Sensitive – Nhạy cảm

**Bảng 2:** Giá trị độ nhạy, độ đặc hiệu của các thuốc lao bằng kỹ thuật giải trình tự trực tiếp từ mẫu đờm

Tên thuốc	Se (%)	Sp (%)	PPV (%)	NPV (%)	P
Isoniazid (INH)	91,70	100,00	100,00	40,00	0,035
Rifampicin (RIF)	86,70	25,00	81,30	33,30	0,109
Ethambutol (EMB)	89,50	68,40	73,90	86,70	0,092
Pyrazinamide (PZA)	67,70	85,70	95,50	37,50	0,094
Streptomycin (SM)	56,50	100,00	100,00	60,00	0,089
Moxifloxacin (MFX)	50,00	96,40	83,30	84,40	0,107
